# The value of seroprevalence data as surveillance tool for Lyme borreliosis in the general population: the experience of Belgium

**DOI:** 10.1186/s12889-019-6914-y

**Published:** 2019-05-17

**Authors:** Tinne Lernout, Benoît Kabamba-Mukadi, Veroniek Saegeman, Marie Tré-Hardy, Morgane de Laveleye, Tommi Asikainen, Ram Benny Dessau, Sophie Quoilin, Amber Litzroth

**Affiliations:** 1 Scientific Directorate Epidemiology and Public Health Sciensano, Juliette Wytsmanstraat 14, 1050 Brussels, Belgium; 2 Institute of Experimental and Clinical Research (IREC), Microbiology Department, Catholic University Louvain (UCL), Brussels, Belgium; 3 Laboratory of Clinical Virology, University Hospitals Leuven, Leuven, Belgium; 4 Department of Medical Microbiology, Laboratoires LBS, Cerba HealthCare, Brussels, Belgium; 5 Department of Clinical Microbiology, Slagelse Hospital, Region Sjælland, Denmark

**Keywords:** Lyme borreliosis, Seroprevalence, Surveillance

## Abstract

**Background:**

Serological surveillance, based on the measurement of the presence of specific antibodies in a given population, can be used in addition to traditional and routine disease surveillance methods. The added value of this has been largely documented for vaccine-preventable diseases, but to a lesser extent for vector-borne diseases. This study aimed to evaluate the utility of seroprevalence data as additional source of information on the epidemiology of Lyme borreliosis in Belgium.

**Methods:**

In total, 3215 residual blood samples collected in 2013–2015 were analysed with Liaison® *Borrelia* IgG kit (DiaSorin S.p.A, Saluggia, Italy). Positive and equivocal results were further examined with immunoblotting (*recom*Line *Borrelia* IgG kit, Mikrogen, Neuried, Germany). Crude prevalence estimates of equivocal and seropositive results were calculated and further adjusted accounting for clustered sampling and standardized for age, sex and population per province, according to the Belgian population structure in 2014. The effect of age, sex and region on seropositivity was assessed using log-binomial regression.

**Results:**

The overall weighted national seroprevalence for *Borrelia burgdorferi* sensu lato, adjusted for clustered sampling, age, sex and province was 1.06% (95%CI 0.67–1.67). Although not statistically significant, the highest prevalences were observed in men and in those younger than 15 years or older than 59 years of age. At provincial level, the seroprevalence estimates do not follow the geographical distribution of tick bites and diagnoses of Lyme borreliosis as detected through other surveillance systems.

**Conclusions:**

Although the use of residual samples for seroprevalence estimates has several advantages, it seems to be a limited tool for serological surveillance of Lyme borreliosis in Belgium, other than follow-up of trends if repeated over time. A population-based sampling strategy might provide a more representative nationwide sample, but would be very time intensive and expensive. Seroprevalence studies within risk groups or risk areas in Belgium could provide a useful alternative approach to complement routine surveillance data of Lyme borreliosis.

## Background

Infectious disease surveillance aims to assess the size of a health problem (disease burden), to identify high risk groups and/or areas to target interventions, to monitor trends and to detect outbreaks, in order to guide public health practice. In addition to routine disease surveillance through physicians and laboratories, serological surveillance, based on the measurement of the presence of specific antibodies in a given population, is frequently used to monitor levels of immunity to or presence of particular diseases within different age groups [1]. This is particularly relevant for vaccine-preventable diseases, for example in the context of measles and rubella elimination targets, where population based seroprevalence studies provide important data on gaps in population immunity and the potential for future outbreaks [2]. For other communicable (non vaccine-preventable) diseases, seroprevalence studies allow the measurement of the occurrence of the disease and associated risk factors. Serosurveys can also be used to assess the intensity of transmission of mosquito borne diseases and to measure the magnitude of an outbreak, like has been shown for dengue [3–5]. In an overview of surveillance strategies for Lyme borreliosis by van den Wijngaard et al., serosurveillance is proposed as a possible surveillance scenario [6].

In Belgium, residual serum samples from laboratories spread over the country were collected between 2013 and 2015, to constitute a serum bank assumed to be representative for the Belgian population. The use of residual samples provides several advantages: they are easily accessible, cheaper and less resource intensive to collect than with population-based sampling. The purpose of the serum bank was to study the seroprevalence of multiple infectious diseases over a 5-year period, with a particular focus on vaccine-preventable diseases. In 2015, the serum bank was used to assess the seroprevalence of antibodies to *Borrelia burgdorferi* sensu lato (s.l.), as an indicator of the lifetime risk for Lyme borreliosis in Belgium.

Lyme borreliosis is a multisystem infectious disease caused by infection with spirochetes of the *B. burgdorferi* s.l. complex. These spirochetes are transmitted to humans through the bite of infected ticks. The disease is the most common tick-borne disease in Europe [7]. Although persons of all ages are at risk for infection, surveillance data suggest that most cases occur in children and elderly persons [7]. Clinical manifestations of infection may include dermatological, rheumatologic, cardiac and/or neurological symptoms, but infection is often asymptomatic. In prospective studies, antibody reactivity to *B. burgdorferi* s.l. (IgG seroconversion) after a tick bite in people without clinical symptoms was observed in 2.9 to 3.7% persons [8–12].

In Belgium, two sources contribute to routine surveillance of Lyme borreliosis. A network of sentinel laboratories performs laboratory surveillance by weekly reporting the number of positive serological tests for *B. burgdorferi* s.l.. And the yearly number of persons hospitalized for Lyme borreliosis is monitored through the hospitals’ minimum clinical datasets. In addition, the incidence of erythema migrans (EM) is estimated based on repeated studies carried out by a sentinel network of general practitioners. Up to 2017, none of the mentioned surveillance sources identified a significant increase in the incidence of Lyme borreliosis [13, 14].

The aim of this study was to evaluate the utility of seroprevalence data as an additional source of information on the epidemiology of Lyme borreliosis in Belgium.

## Methods

### Blood sample collection

A cross-sectional study design was used to constitute a serum bank, representative of the general population living in Belgium. Between July 2013 and January 2015, residual sera were collected through voluntary participating diagnostic laboratories that are part of the Belgian sentinel laboratory network. To avoid (over) selection of immunosuppressed and severely or chronically ill subjects, only specimens from surgery, orthopaedic, emergency and otorhinolaryngology hospital wards and from ambulatory diagnostic laboratories were collected. The total number of specimens to be collected was estimated at 3600, based on sample size estimations of the European Sero-Epidemiology Network (ESEN) and previous experience with age-specific analyses of seroprevalence data in Belgium [1, 15, 16]. To allow for geographical representativeness at regional and provincial level, each participating laboratory was allocated a fixed number of specimens, based on the population density in the laboratory’s region (using kernel smoothing) and the number of participating laboratories in the area. The total number of specimens per laboratory (ranging from 105 to 210) was further stratified by sex and by age groups.

Since residual samples were used, the only data available at laboratory level for each sample were date of sampling, date of birth, sex and postal code of residence.

### Laboratory methods

As a serological marker for past infection with *B. burgdorferi* s.l., serum specific IgG antibodies were used. Seropositivity indicates (historical) exposure to the agent and not necessarily (past) clinical disease. Laboratory testing was performed by the Belgian National Reference Centre for *B. burgdorferi* s.l. (Catholic University Louvain, microbiology department), according to the standard operating procedures. In line with the recommendations for serological confirmation of clinical cases, a two-tier testing algorithm was used [17]. In a first step, the commercially available Liaison© *Borrelia* IgG kit (DiaSorin S.p.A, Saluggia, Italy) was used. This kit uses a chemiluminescence immunoassay (CLIA) technology for the quantitative determination of specific IgG to *B. burgdorferi* s.l., using the recombinant *Borrelia* VlsE antigen [18]. The diagnostic performance of DiaSorin’s immunoassay has already been extensively evaluated in the literature [18–21]. An evaluation of the assay on 180 blood samples from both patients (with Lyme borreliosis and other diseases) and healthy blood donors in Belgium reported a diagnostic sensitivity for IgG results of 100% and a specificity of 91.4% [18]. In the second step, positive CLIA IgG positive (> 15 UI) and equivocal results (> 10 UI and ≤ 15 UI) were further confirmed by immunoblotting, using the *recom*Line *Borrelia* IgG kit (Mikrogen, Neuried, Germany), a strip-immunoassay with antigens produced by recombinant techniques. The IgG sensitivity and specificity of this kit in the Belgian study by Busson et al. was 100 and 98% respectively [18]. When applying the 2-step strategy (DiaSorin immunoassay followed by *recom*Line *Borrelia* IgG kit), no false positive nor false negative results were observed in the study [18].

### Data analysis

Crude prevalence estimates of equivocal and seropositive results were calculated and further adjusted accounting for clustered sampling and standardized for age, sex and population per province, according to the Belgian population structure in 2014. Equivocal results in the second tier (immunoblotting) were included as positives in the final analyses. Weighted results (adjusted prevalence ratios) with their 95% confidence intervals (CI) are presented. When the crude number of observations was equal to zero, the estimate of the upper limit of the confidence interval was calculated as 1-(0.05)^(1/n), where n is the sample size. Pearson’s Chi-squared (χ^2^) and Fisher’s exact test were used to compare proportions. The effect of age, sex and region on seropositivity was assessed using log-binomial regression. Variables that were significant (*p* < 0.10) in univariate analysis were included in the multivariate model. *P* values < 0.05 were considered statistically significant in the multivariate model. The effect of age, sex and region was also estimated on the continuous quantitative relative light units (RLU) results. We used STATA 13 (Statcorp College Station, TX, USA) for statistical analyses.

## Results

Of a total of 160 diagnostic laboratories in Belgium in 2013, 28 (17.5%) participated to the collection of serum samples. Between July 2013 and January 2015, 3257 specimens were collected, of which 3215 could be used (sufficient volume available) for testing for *B. burgdorferi* s.l. antibodies. Of these specimens, 378 (11.8%) were from individuals living in the region of Brussels, 785 (24.4%) from the Walloon region and 2052 (63.8%) from the Flemish region. The sex ratio was 1 (1608 women versus 1607 men).

**Fig. 1:**
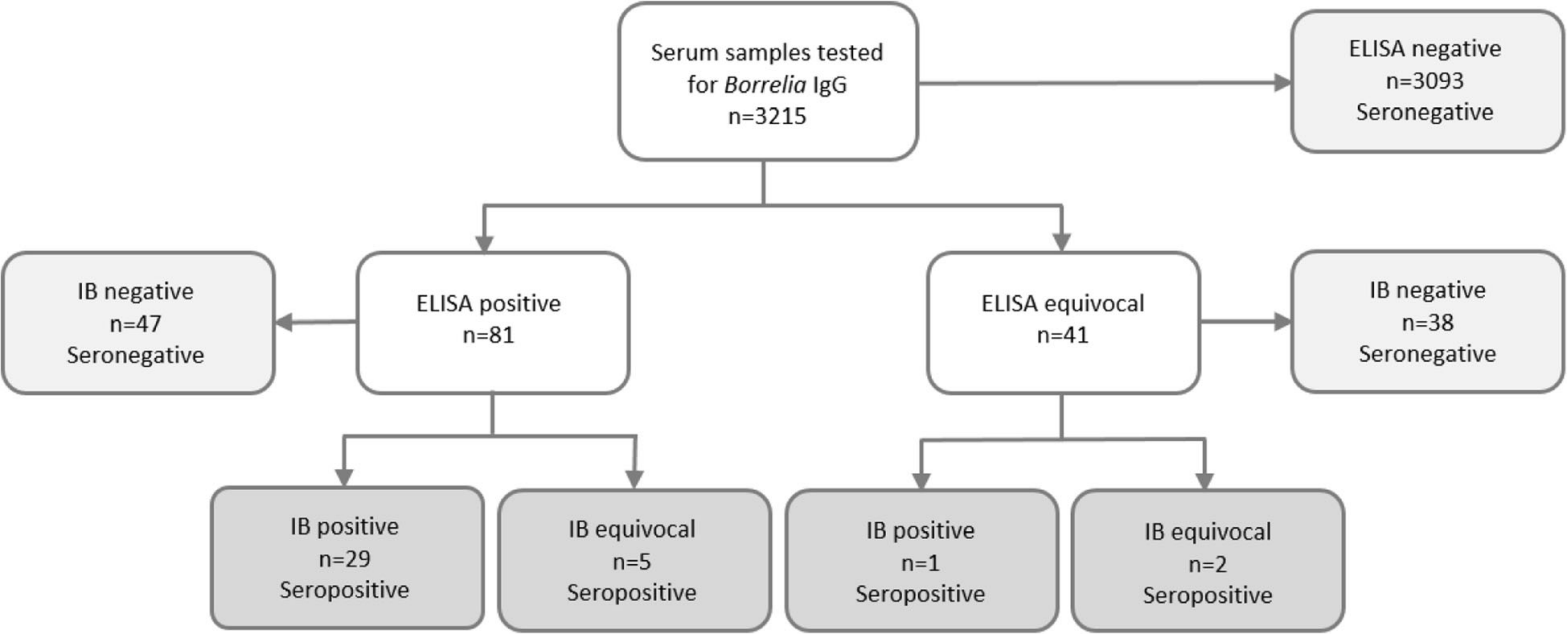
Flow chart of samples tested, according to the CLIA and Immunoblotting (IB) results

Out of the 3215 specimens tested for *B. burgdorferi* s.l. IgG with CLIA testing, 3093 (96.2%) were seronegative, 41 (1.3%) had an equivocal result and 81 (2.5%) were positive (Fig. 1). Further testing of the equivocal and positive samples with immunoblot resulted in 30 positive specimens (0.9%) and 7 equivocal (0.2%).

The overall weighted national seroprevalence for *B. burgdorferi* s.l., adjusted for clustered sampling, age, sex and province, was 1.06% (95%CI 0.67–1.67). Based on the CLIA screening test only, it was 3.91% (95%CI 3.08–4.96).

The highest prevalences were observed in men and in those younger than 15 years and those older than 59 years of age (Table 1). However, these differences were not statistically significant.

**Table 1:** Adjusted prevalence of *Borrelia* IgG seropositivity and 95% confidence intervals by sex, age and region

Characteristic	Number positive^a^ samples/total number	Adjusted prevalence %	95% CI	*P* value
Sex				
Male	24/1608	1.34	0.81–2.20	
Female	13/1607	0.78	0.32–1.90	0.29
Age (in years)				
< 15	12/1271	1.16	0.62–2.16	
15–59	18/1590	0.91	0.50–1.63	0.94
≥ 60	7/354	1.38	0.48–3.89	0.58
Region				
Brussels	4/378	1.21	0.36–4.02	
Flanders	27/2052	1.12	0.68–1.82	0.91
Wallonia	6/785	0.93	0.31–2.74	0.75
Province				
West Flanders	2/522	0.44	0.00–2.33	
East Flanders	2/431	1.23	0.31–4.77	0.34
Flemish Brabant	8/532	1.06	0.39–2.84	0.37
Antwerp	7/551	0.79	0.34–1.86	0.54
Limburg	12/394	2.65	1.25–5.53	0.03^b^
Hainaut	0/298	0	0.00–0.01	0.37
Walloon Brabant	2/69	1.12	0.27–4.46	0.39
Liège	3/175	2.82	0.78–9.72	0.05
Namur	0/62	0	0.00–0.05	0.64
Luxembourg	1/181	0.53	0.00–3.67	0.89

^a^Both the 30 positive and 7 equivocal IB results were considered as positive samples. ^b^Statistically significant

Differences in distribution of RLU values by age, gender and province were not statistically significant neither (results not shown)

Likewise, there were no statistically significant differences observed in prevalence by region nor by province, except for the province of Limburg (in eastern Belgium), where a higher prevalence was registered compared to the province of West Flanders (in western Belgium), 2.65% (95%CI 1.25–5.53) versus 0.44% (95%CI 0.00–2.33) (Table 1). In this case the 95% confidence intervals overlap but performing Pearson X^2^ -test showed a statistical difference (*p* = 0.03) in estimated prevalence.

## Discussion

Seroprevalence data provide information on the cumulative incidence of exposure to *B. burgdorferi* s.l. in a certain geographical area. In Belgium, an overall weighted seroprevalence for *B. burgdorferi* s.l. of 1.06% (95%CI 0.67–1.67) was observed after two-tier testing. The weighted seroprevalence after the screening test was 3.91% (95%CI 3.08–4.96).

Our results are in line with those of a smaller study in blood donors in both a rural (*n* = 209) and an urban area (*n* = 193) in Belgium in 2011 [22]. In that study, 4.3 and 3.1% of the donors respectively presented a positive or equivocal result for *Borrelia* IgG based on CLIA testing (Liaison XL) only, compared with our estimate of 3.9% using the same test. A smaller study on 50 blood samples collected from healthy blood donors in 2007–2009 reported an IgG seroprevalence of 2% [18]. And in an older study in the early nineties on 1916 young military blood donors, the seroprevalence of borderline and positive results was 3.2%, using an in-house Elisa kit [23]. Our results are also comparable to an estimated 4.7% prevalence based on C6-ELISA only as was reported in a study in a healthy population (*n* = 836) in our neighbouring country the Netherlands [24]. The slightly higher estimate in the Netherlands is in line with a higher incidence of erythema migrans diagnosed by general practitioners, with 140 diagnoses per 100,000 inhabitants in 2014 [25] compared to 97.6 per 100,000 in Belgium per year in 2015–2017 [14].

Studies in other European countries using two-tier testing in various study populations report a wide variation of prevalence results, with estimates ranging from 1.6% in blood donors in Slovakia up to 9.4% in healthy adults in Germany [26–30]. However, seroprevalence estimates should be compared with caution, as the representativeness of the population covered and laboratory testing methods differ between studies.

Although the difference in prevalence observed by sex is not statistically significant in our study, a higher seroprevalence in men is also observed in studies in other countries [27, 28, 30]. This is likely due to a higher exposure to ticks during professional and leisure activities. Likewise, although not statistically significant in our study, an increased risk for infection is generally observed among older persons (> 59 years of age), reflecting the population’s cumulative exposure to *B. burgdorferi* s.l. [27, 30].

Based on the results of Lyme borreliosis surveillance and the geographical occurrence of tick bites reported through an online citizen-based platform (TiquesNet), the risk of getting a tick bite or developing Lyme borreliosis in Belgium is higher in the provinces of Luxemburg, Flemish and Walloon Brabant, Limburg, Antwerp and Namur [13, 31]. This is not reflected in our study, where the prevalence was significantly higher in the province of Limburg compared to the western part of the country only. Although trying to have geographical representativeness by allocating a fixed number of specimens to each laboratory and further correcting crude prevalence estimates for cluster sampling, specific hot spots for Lyme borreliosis might not have been reached by sampling. This is not surprising, since the distribution of ticks and the pathogens they can be infected with are heavily influenced by spatial factors (landscape composition and configuration, forest and wildlife management, host abundance), that can have important local variation [32, 33]. Although Belgium is a small country, the topography is very different from West to East, with a coastal flat region gradually changing over a central plane to a heavily wooded region in the east and southeast of the country. Even within provinces, the landscape can be diverse, with focal spots of suitable habitat for ticks. The sample collection method used for the current serum bank (based on residual samples) might thus not be suitable to study the seroprevalence in the general population of diseases with strong environmental determinants.

Using residual samples also has some general limitations. The information available for each sample is basic and data on possible risk factors cannot be collected. In addition, there might be a possible selection bias: serum specimens submitted to diagnostic laboratories may not be entirely representative of the general population, in particular for a disease like Lyme borreliosis, most often contracted during physical activities. Studies on vaccine preventable diseases have shown that a convenience sample of sera produces similar estimates of immunity to those obtained from a sample collected using a randomized cluster design [34, 35]. To our knowledge, a similar comparison has not been performed for other diseases. To limit the possible bias in our study and exclude people with chronic diseases and immune disorders, only samples from selected medical wards have been included. Moreover, our results are in line with estimates of the prevalence of *Borrelia* antibodies in blood donors (both from a rural or urban area) in Belgium. Generally, blood donors are expected to be less prone than average to chronic disorders and autoimmune diseases, given the permanent and temporary exclusion criteria for blood donors, as well as other confounding factors related to lifestyle resulting in a selection of healthier and more health-conscious persons [36]. Therefore, we believe that an oversampling of an ill low-risk population is unlikely to have occurred.

Using seroprevalence data for surveillance of Lyme borreliosis specifically presents other limitations. The results only provide information on historical exposure to *B. burgdorferi* s.l. but not on the incidence of disease, as asymptomatic infections do occur [27, 28]. Also, although the humoral immune response to an infection with *B. burgdorferi* s.l. is often long lasting, the persistence of antibodies can vary widely, going from several months to many years [37–39]. A seroprevalence of 1% is therefore not an exhaustive estimation of exposure to the bacteria.

Finally, serological tests for Lyme borreliosis have their own limitations, widely discussed in the scientific literature [18, 40–42]. They include the complexity of the antigen composition of *B. burgdorferi* s.l., cross-reactivity of *B. burgdorferi* antigens leading to false positive results and the lack of a clear European consensus on criteria of the specific bands required to be positive among the second-tier commercial immunoblotting kits. In our study, both equivocal and positive results after the confirmatory immunoblot were considered as positives, since the specificity of being both ELISA positive and at least equivocal in second tier is considered very high. This is possibly different in the clinical situation where the confirmation of patients with active disease, especially Lyme arthritis and acrodermatitis, would preferably require more antigens to be positive in order to distinguish the natural background seroreactivity [43]. A review by Leeflang et al. observed an important heterogeneity in sensitivity and specificity of different commercial and in house serological assays for Lyme borreliosis in Europe [44]. And a study by Ang et al. on the influence of assay choice on the results in a two-tier testing algorithm for the detection of anti-Borrelia antibodies concluded that the choice of ELISA-immunoblot combination severely influences the number of positive results, making the exchange of test results between laboratories with different methodologies hazardous [42]. In order to allow comparison of the results of our study with previous (smaller) studies in Belgium, the same serology kit was used. The results of Busson et al. suggested high performance of *Borrelia* IgG screening (Liaison® Borrelia IgG) and confirmatory (*recom*Line Borrelia IgG) kits in Belgium [18].

## Conclusions

Seroprevalence studies on residual samples have previously been validated for the study of immunity of vaccine preventable diseases, but not for diseases that are strongly influenced by environmental determinants such as tick-borne diseases. The serum bank that was constituted in Belgium for serosurveys on vaccine preventable diseases mainly seems to have a limited added value for surveillance of Lyme borreliosis, other than a follow-up of the exposure to *B. burgdorferi* s.l. over time if the study is repeated every 3–5 years using the same methodology and testing.

The main conclusion of our study is that the overall exposure to *B. burgdorferi* s.l. in the general population in Belgium is low; 99% of the general population has no antibodies. This implies that in the absence of other evidence supporting a Lyme borreliosis diagnosis, a person presenting with non-specific or subjective symptoms for more than six weeks with a negative serologic test result in Belgium would strongly support an alternate diagnosis [43].

However, the observed prevalence of the disease is too low to detect regional differences, even at the level of provinces (second geographical level in Belgium). A population-based sampling taking into account environmental determinants for exposure to tick bites would allow collecting a more representative sample for a study on *B. burgdorferi* s.l. exposure than a survey based on residual sampling. But this is a very time intensive and expensive method. Seroprevalence studies in risk groups or risk areas in Belgium could be a useful alternative approach to better target populations for prevention.

## Abbreviations

*B. burgdorferi* s.l: *Borrelia burgdorferi* sensu lato
CI: Confidence interval
CLIA: Chemiluminescence immunoassay
EIA: Enzyme immunoassay
EM: Erythema migrans
IB: Immunoblotting
